# A Novel Germline *MUTYH* Mutation (p.W156∗) in High-Grade Astrocytoma, *IDH* Mutant

**DOI:** 10.1155/humu/4321571

**Published:** 2025-05-28

**Authors:** Lulu Zhang, Shaoyan Xi, Lei Yuan, Ziteng Li, Xiaoyun Liu, Jiamei Gu, Shuo Li, Liyun Huang, Wanming Hu, Lingyi Fu

**Affiliations:** ^1^State Key Laboratory of Oncology in South China, Guangdong Provincial Clinical Research Center for Cancer, Sun Yat-sen University Cancer Center, Guangzhou, China; ^2^Department of Molecular Diagnostics, Sun Yat-sen University Cancer Center, Guangzhou, China; ^3^Department of Pathology, Sun Yat-sen University Cancer Center, Guangzhou, China

**Keywords:** astrocytoma, germline mutation, *IDH*, *MET*, *MUTYH*

## Abstract

Germline mutations in the DNA repair gene *E. coli MutY homolog* (*MUTYH*) are established predisposing factors for colorectal polyposis, colorectal carcinoma, and various extracolonic malignancies. Nevertheless, the association between *MUTYH* mutations and central nervous system (CNS) tumorigenesis remains poorly characterized. In this study, we reported the first identification of a novel c.467G > A (p.W156∗) *MUTYH* variant in two patients with high-grade astrocytoma, *IDH mutant*, which was classified as pathogenic. Histopathological evaluation revealed tumor morphologies consistent with either diffuse glioma or giant cell glioblastoma. Comparative analysis with mismatch repair (MMR)–deficient tumors demonstrated that patients carrying *MUTYH* mutations exhibited microsatellite stability, relatively low tumor mutation burden (TMB), and an immunosuppressive microenvironment, indicating difficulties in benefiting from immunotherapy. Fortunately, gain of Chromosome 7, in association with amplification of the *MET* gene, was detected, underscoring the possible application of targeted drugs. Integrating previous studies, we summarized germline *MUTYH* mutations in 11 cases of high-grade neuroepithelial tumors (eight gliomas and three medulloblastomas). This cohort demonstrated a predilection for pediatric and young adult populations without significant gender predominance. Our findings suggested a potential association between germline *MUTYH* mutations and CNS tumor susceptibility.

## 1. Introduction

The *E. coli MutY homolog* (*MUTYH*, also named *MYH*) gene is involved in DNA repair via the base excision repair (BER) system [1]. It encodes a DNA glycosylase that prevents G:C → T:A transversions induced by guanine oxidation [2]. Germline mutations of *MUTYH* are associated with multiple colorectal polyposis or adenomas, termed *MUTYH*-associated polyposis (MAP). MAP follows an autosomal recessive pattern of inheritance and causes an increased lifetime risk of colorectal cancer (CRC) [3, 4]. In contrast, somatic mutations of *MUTYH* are not implicated in CRC pathogenesis [5]. Patients with MAP also exhibit a significantly elevated incidence of extracolonic malignancies, including ovarian, bladder, skin, adrenal gland, pancreatic, and thyroid cancers [6, 7]. Both biallelic (compound heterozygous or homozygous) and monoallelic (heterozygous) mutations of *MUTYH* are reported to be pathogenic. However, the former confers a substantially higher risk of colorectal and extracolonic cancers, while the latter may trigger tumorigenesis upon loss of the functional allele [8].

The risks of CNS tumors for *MUTYH* mutation carriers are not well defined. Kline et al. first described inactivating germline mutations of *MUTYH* in two pediatric patients with high-grade gliomas (one glioblastoma with Histone H3 K27M–mutant protein expression and one medulloblastoma) in 2016 [9]. Subsequently, Bedics et al. identified germline *MUTYH* mutations in two cases of pediatric Histone H3–mutant gliomas (one diffuse hemispheric glioma, *H3 G34* mutant, and one diffuse midline glioma, H3 K27 altered) [10]. In 2023 and 2024, Villy et al. and Cipri et al. sequentially reported two cases of medulloblastoma harboring pathogenic germline *MUTYH* variants [11, 12]. McDonald et al. also identified germline mutations of *MUTYH* in three of 152 patients with adult-type diffuse gliomas [13]. Furthermore, some reports described *MUTYH* mutations in pilomyxoid astrocytoma, ependymoma, and atypical central neurocytoma, though the mutational origin (germline or somatic) remained unconfirmed [14, 15].

Here, we reported two cases of high-grade astrocytoma, *IDH* mutant in young adults harboring germline *MUTYH* mutations. A novel pathogenic c.467G > A (p.W156∗) *MUTYH* variant was detected. In addition to the *MUTYH* mutation, both patients exhibited gain of Chromosome 7, which was associated with *MET* gene amplification. Integrating previous studies, we summarized a total of 11 high-grade glioma cases with germline *MUTYH* mutations. Clinical and follow-up details were available in eight of these patients. This tumor predominantly affected pediatric and young adult populations without sex predilection. Two pediatric patients succumbed to the disease, and five of the patients experienced tumor recurrence. Our findings suggested a potential association between germline *MUTYH* mutations and CNS tumor susceptibility.

## 2. Materials and Methods

### 2.1. Collection of Tumor Specimens and Clinical Data

The cases were collected from Sun Yat-sen University Cancer Center over a 3-year period (2021–2024). The clinical features and follow-up details were extracted from medical records. The histology of these cases was reviewed, and extended immunohistochemistry (IHC) and next-generation sequencing (NGS) panels were utilized to confirm the diagnosis of “astrocytoma, *IDH* mutant.”

Progression-free survival (PFS) was defined as the duration from diagnosis to tumor recurrence or death, and overall survival (OS) was defined as the duration from diagnosis to death from any cause.

Written informed consent was obtained from the patients or their legal representatives. Patient identities were anonymized. Ethical approval was granted by the Sun Yat-sen University Cancer Center Institute Research Ethics Committee, and all procedures adhered to the International Ethical Guidelines for Biomedical Research Involving Human Subjects (CIOMS).

### 2.2. IHC Analysis

All cases were processed as formalin-fixed and paraffin-embedded (FFPE) specimens with sections cut to 4–5 *μ*m. Subsequently, the sections were subjected to heat-induced epitope retrieval using pH 6.0 buffer at 100°C for 20 min. All IHC procedures were performed using a Leica Bond III Biosystem automated staining system. The following primary antibodies were used: glial fibrillary acidic protein (GFAP) (clone UMAB129, ZM-0118, ZSGB-BIO, Beijing, China), Oligodendrocyte Transcription Factor 2 (Olig2) (clone EP112, ZA-0561, ZSGB-BIO), IDH1 R132H (clone OTI3G9, ZM-0447, ZSGB-BIO), alpha-thalassemia/mental retardation syndrome X-linked protein (ATRX) (ZA-0016; ZSGB-BIO), H3K27M (clone RM192, ZA-0321, ZSGB-BIO), H3K27me3 (clone RM175, ZA-0327, ZSGB-BIO), CD68 (clone KP1, ZM-0060, ZSGB-BIO), CD163 (clone 10D6, ZM-0428, ZSGB-BIO), CD4 (clone EP204, ZM-0519, ZSGB-BIO), CD8 (clone SP16, ZM-0508, ZSGB-BIO), and Ki-67 (clone MIB-1, ZM-0167, ZSGB-BIO).

### 2.3. Molecular Analysis

For NGS and molecular analysis, we followed the methods of our previous study [16]. Sequencing was performed on the Illumina NextSeq platform, using 300-cycle paired-end sequencing. Subsequently, the sequencing data were processed and analyzed. A custom pipeline was developed to perform read alignment, variant calling, fusion detection, copy number variation (CNV) identification, and quality control. The fastp program (v2.20.0; https://github.com/OpenGene/fastp) was used for adapter trimming. Sequence reads were aligned against the human reference genome (hg19) using the BWA-mem aligner (v.0.7.17; https://github.com/lh3/bwa), with additional realignment of select regions using ABRA2 (v2.21; https://github.com/mozack/abra2). Candidate tumor-specific mutations, consisting of point mutations, small insertions, and deletions, were identified and annotated using VarDict (v1.5.7; https://github.com/AstraZeneca-NGS/VarDict) and InterVar (https://github.com/WGLab/InterVar). CNVs and fusions were analyzed using CNVkit (dx1.1; https://github.com/etal/cnvkit) and FACTERA (v1.4.4; https://github.com/FredHutch/easybuild-life-sciences/tree/main/fh_easyconfigs/f/factera), respectively. Additional filtering and inspection of somatic mutations, CNVs, and fusion results were performed using custom scripts. CNV burden was defined as the total length of autosomal CNVs divided by all autosomal lengths.

Paired blood samples were submitted with tumors to determine the germline variants. Germline variants were classified as pathogenic, likely pathogenic, uncertain significance, likely benign, or benign based on guidelines put forth by the American College of Medical Genetics and Genomics (ACMG) [17, 18].

Methylguanine methyltransferase (*MGMT*) promoter methylation was assessed by qPCR using the *MGMT* gene methylation kit (Sinomdgene, Beijing, China) according to the manufacturer's instructions.

### 2.4. Literature Review

A thorough literature search was performed on PubMed (http://www.ncbi.nlm.nih.gov/pubmed/) to identify previously reported cases with germline mutations of *MUTYH*. Different combinations of keywords, including “MUTYH,” “MYH,” “MAP,” “germline mutation,” “glioma,” “IDH”, “MET,” and “CNS,” were used in the title/abstract field. Overlapping cases from multiple papers were combined for analysis.

### 2.5. Statistical Analysis

The *χ*^2^ test was used to compare categorical variables, and survival analysis was performed using the Kaplan–Meier method, with comparison made using the log-rank test. Survival time was measured in months from diagnosis. Statistical analysis was performed using SPSS 26.0 software, and significance was set at a *p* value < 0.05.

## 3. Results

### 3.1. Clinicopathological Characteristics

#### 3.1.1. Case 1

**Figure 1:**
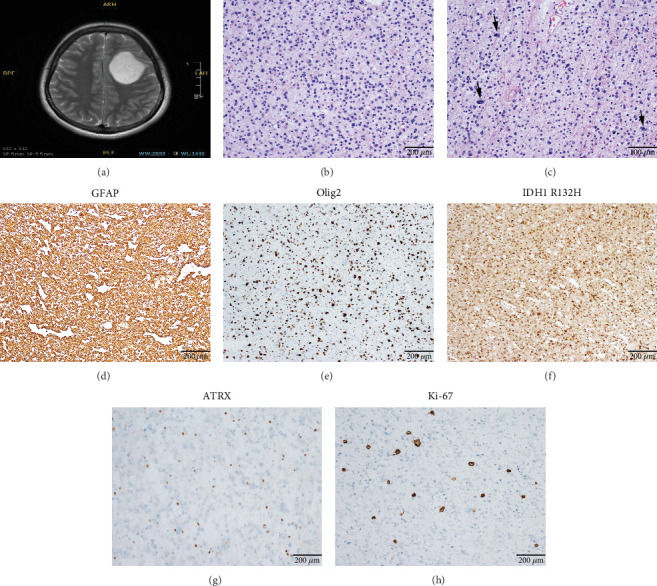
Clinical and histopathological characteristics of Case 1. (a) MRI revealed the lesion in the left frontal lobe. (b) The morphology of high-grade diffuse glioma (100×). (c) Hypercellularity and increased proliferative activity (200×). (d) Immunoreactivity for GFAP (100×). (e) Immunoreactivity for Olig2 (100×). (f) Strong and diffuse positive staining for IDH1 R132H (100×). (g) The expression of ATRX was absent (100×). (h) The Ki-67 labeling index was about 8% (100×).

A 30-year-old female presented with convulsions of the limbs and headache for 5 days, without fever, nausea, or vomiting. MRI revealed a 54∗41-mm mass in the left frontal lobe containing solid and cystic components (Figure 1a). Surgical resection successfully achieved total tumor removal. Following surgery, she received concurrent radiochemotherapy (temozolomide). At the 3-month postoperative follow-up, she experienced an uneventful recovery without neurological deficits. MRI showed no signs of recurrence. The patient received surgical resection for “fibroadenoma of the breast” 10 years ago. She and her family had no history of colorectal polyposis or cancer or extracolonic malignancies. Her family members refused germline genetic testing.

Microscopically, this case presented the morphology of diffuse glioma, composed of poorly differentiated astrocytes with abundant eosinophilic cytoplasm and oval hyperchromatic nuclei (Figure 1b). It was classified as CNS WHO Grade 3 due to hypercellularity and increased proliferative activity without tumoral necrosis or microvascular proliferation (Figure 1c). Tumor cells typically showed strong immunoreactivity for GFAP (Figure 1d) and Olig2 (Figure 1e). IDH1 R132H staining was strongly and diffusely positive (Figure 1f). The expression of ATRX was lost (Figure 1g). The Ki-67 labeling index was approximately 8% (Figure 1h).

#### 3.1.2. Case 2

**Figure 2:**
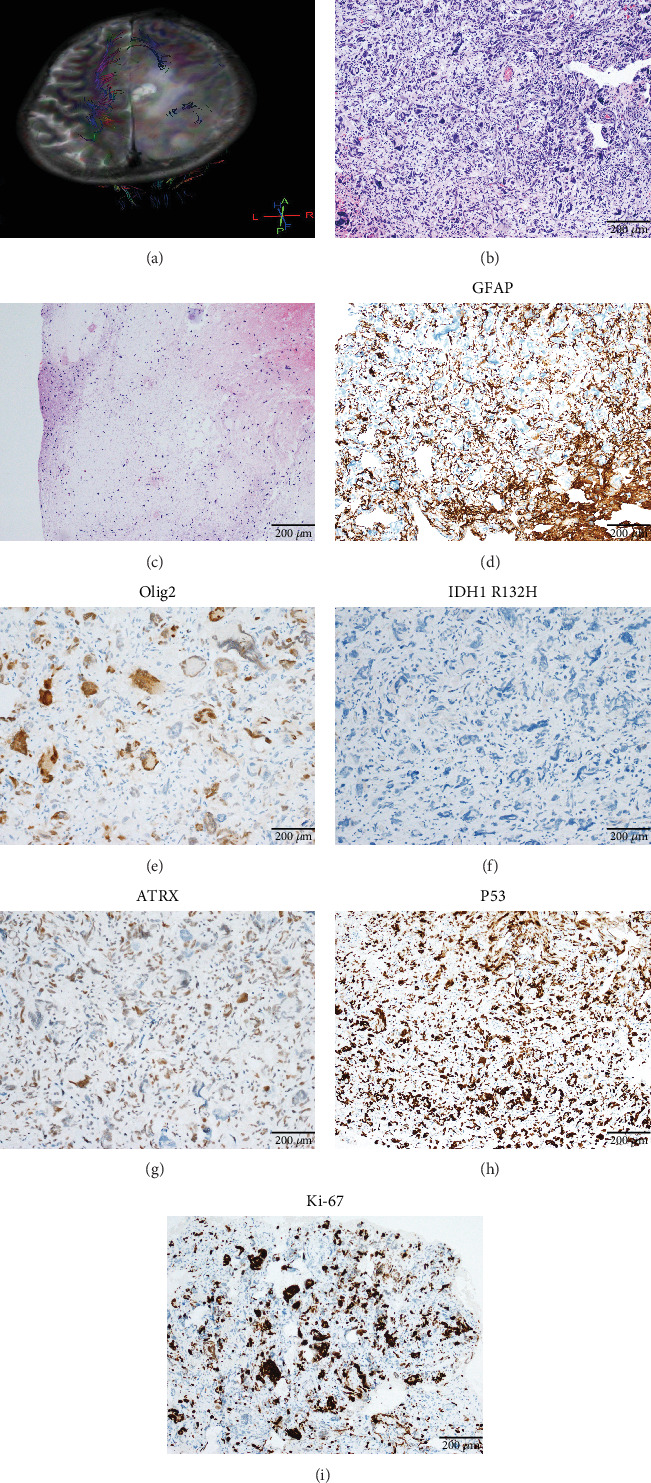
Clinical and histopathological characteristics of Case 2. (a) MRI revealed the lesion in the right frontal lobe. (b) The morphology of giant cell glioblastoma (100×). (c) Patchy tumoral necrosis (100×). (d) Immunoreactivity for GFAP (100×). (e) Immunoreactivity for Olig2 (100×). (f) IDH1 R132H was negative (100×). (g) The expression of ATRX was retained (100×). (h) Strong and diffuse positive staining for P53 (100×). (i) The Ki-67 labeling index was about 40% (100×).

A 38-year-old male presented with paroxysmal convulsions of the limbs, gaitism, and loss of consciousness for 2 days, without fever, nausea, headache, or vomiting. MRI revealed a 19∗14-mm mass in the right frontal lobe containing solid and cystic components (Figure 2a). Surgical resection successfully achieved total tumor removal. Following surgery, he received concurrent radiochemotherapy (temozolomide). However, recurrence of the tumor in the right parietal lobe was detected on MRI during the 2-month follow-up. He is currently hospitalized and preparing for the second surgery. The patient and his family had no history of colorectal polyposis or cancer, as well as extracolonic malignancies. His family members refused germline genetic testing.

Case 2 was histologically diagnosed as “giant cell glioblastoma” (CNS WHO Grade 4) based on prominent multinucleated giant cells and high mitotic activity (Figure 2b). Patchy tumoral necrosis was also observed (Figure 2c). Tumor cells exhibited variable immunoreactivity for GFAP (Figure 2d) and were positive for Olig2 (Figure 2e). IDH1 R132H was negative (Figure 2f). ATRX expression was retained (Figure 2g). Tumor cells demonstrated strong and diffuse positive p53 positivity, indicative of a *TP53* mutation (Figure 2h). The Ki-67 labeling index was approximately 40% (Figure 2i).

### 3.2. Gene Expression Profiling

Mutations in *IDH1* were confirmed by NGS. Case 1 showed a c.395G > A (p.R132H) missense mutation in the *IDH1* gene, while Case 2 carried a c.395G > T (p.R132L) variant. It was consistent with IHC results that IDH1 R132H was positive in Case 1 and negative in Case 2. Neither tumor exhibited whole-arm codeletion of Chromosomes 1p and 19q, supporting the diagnosis of “astrocytoma, *IDH* mutant.”

**Figure 3:**
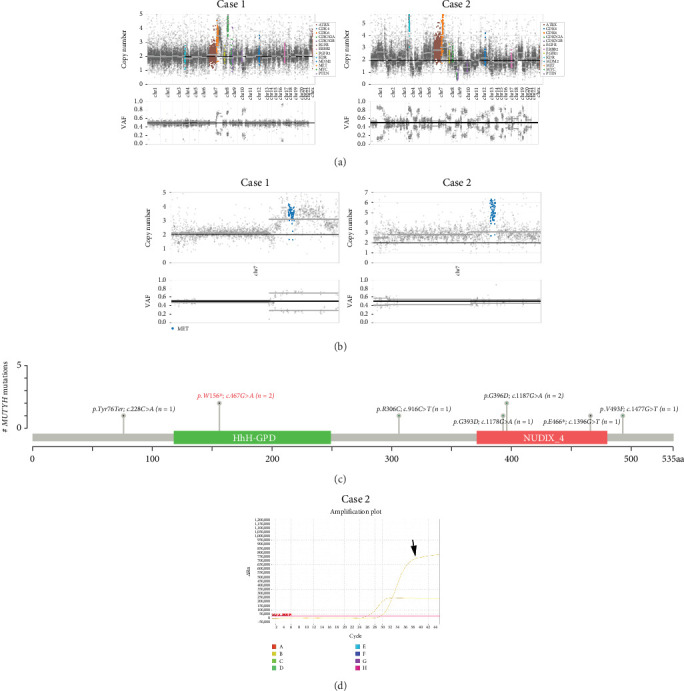
Gene expression profiling. (a) Chromosomal CNVs. (b) Amplification of the *MET* gene. (c) Schematic diagram of *MUTYH* variants. (d) Methylation of the *MGMT* promoter in Case 2.

Chromosomal CNV burden varied between cases (Figure 3a). Both tumors showed gain of Chromosome 7, associated with *MET* gene amplification (Figure 3b). NGS identified a nonsense mutation in Exon 6 of the *MUTYH* gene. Guanine (G) at Position 467 in the coding sequence was replaced by adenine (A), leading to the substitution of tryptophan (W) at Position 156 by a stop codon (∗), finally resulting in a truncated and potentially nonfunctional protein (Figure 3c). This novel c.467G > A (p.W156∗) variant was regarded as pathogenic by bioinformatics analysis. It is known that loss-of-function variants in *MUTYH* are pathogenic (PVS1) [19, 20]. Previous studies have identified this variant in multiple individuals with CRC [21, 22]. The ClinVar database lists it as a pathogenic/likely pathogenic variant (PP5). Its mutation frequency in the Genome Aggregation Database (gnomAD) is extremely low, only 0.000033 (PM2). Based on the comprehensive analysis, this mutation is classified as pathogenic according to ACMG guidelines.

Among 295 patients who underwent brain tumor–targeted NGS in our center, 18 harbored germline *MUTYH* mutations, but only the two described cases carried pathogenic variants. Both tumors were microsatellite stable (MSS) with low tumor mutational burden (TMB: 2.83 mut/Mb in Case 1; 9.43 mut/Mb in Case 2).

Case 1 had a c.4376C > T (p.R1426∗) nonsense mutation in the *ATRX* gene. She also harbored somatic mutations in the *TP53* and *NOTCH1* genes. In Case 2, amplification of *PDGFRA* and *CDK6* and homozygous deletion of *CDKN2A* and *CDKN2B* were detected, while no mutations of *ATRX* were found. He had somatic mutations in other genes such as *TP53*. *MUTYH* was the only germline mutation in these two patients, and other mutations were somatic. Gene alterations affecting *BRAF*, *NF1*, *FGFR*, *NTRK*, *MYB*, *MYBL1*, *PIK3CA*, *PIK3R1*, *PTEN* (*phosphatase and tensin homolog*), *CIC*, *FUBP1*, *MN1*, *ZFTA* (*c11orf95*), *RELA*, *YAP1*, *MYC*, *MYCN*, or *Histone H3* genes were not detected. No *TERT* promoter mutation was observed. Additionally, Case 2 showed methylation of the *MGMT* promoter (Figure 3d). The germline mutation profile of the two cases is provided in Table 1. The detailed summary of all genomic alterations is shown in Table S1.

### 3.3. Immune Microenvironment

**Figure 4:**
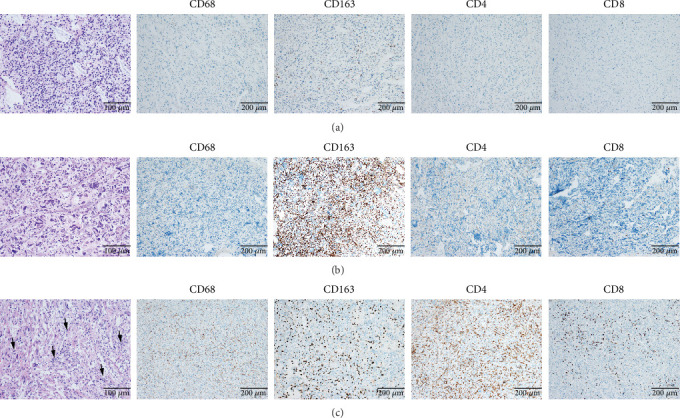
Immune microenvironment. (a) Representative HE and IHC images of Case 1. (b) Representative HE and IHC images of Case 2. (c) Representative HE and IHC images of the case with LS.

As shown in Figure 4a,b, both cases exhibited a paucity of immune cells in the tumor microenvironment. CD163^+^ M2 tumor–associated macrophages (TAMs) predominated, whereas CD4^+^ and CD8^+^ T cells were scarce, indicating an immune-suppressive microenvironment.

For comparison, one patient diagnosed with Lynch syndrome (LS) with a germline mutation of the *MSH2* gene was included as a control. Her right frontal tumor was pathologically diagnosed as “glioblastoma, *IDH* wild type.” Significantly abundant tumor-infiltrating lymphocytes (TILs) were observed. The proportion of CD163^+^ M2 TAMs was lower compared to patients with *MUTYH* mutations (Figure 4c).

### 3.4. Literature Review and Survival Analysis

A thorough PubMed database search identified nine additional cases of germline *MUTYH* mutations in CNS tumors. These cases were retrieved from five articles published between 2016 and 2024 [9–13]. Combined with our two cases, we analyzed the molecular profiles of these 11 cases. Clinical and follow-up details were available for eight of these patients. Pathogenic *MUTYH* variants are summarized in Table 2 and prognostic data in Table 3. All patients presented with CNS-related symptoms as the initial manifestation, but only one had colorectal adenomatous polyposis. None had a history of CRC or extracolonic malignancies.

**Figure 5:**
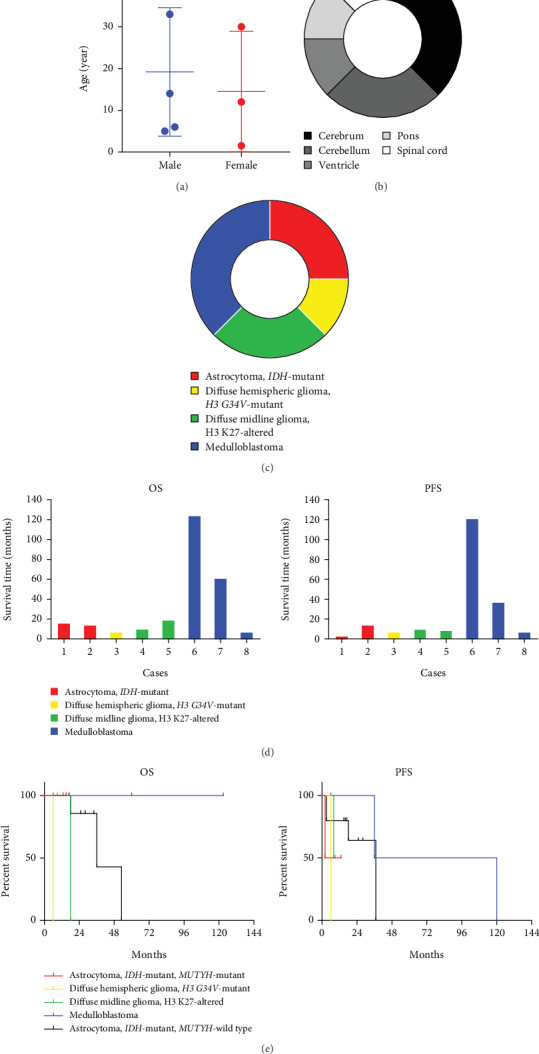
Survival analysis. (a) Age and gender distribution of the patients. (b) Tumor locations. (c) Tumor subtypes. (d) Illustration of survival information. (e) Survival curves.

As illustrated in Figure 5a, high-grade neuroepithelial tumors with germline mutations in *MUTYH* predominantly affected children and young adults, with no sex predominance. Most tumors were intracranial (Figure 5b). Among the eight patients with available follow-up data, five were diagnosed with gliomas and three with medulloblastomas (Figure 5c). Individual patient survival outcomes in column diagrams are presented in Figure 5d. A control cohort (*n* = 10) diagnosed as astrocytoma, *IDH* mutant without *MUTYH* mutations in Sun Yat-sen was included for comparison. Tumor type–stratified survival curves are shown in Figure 5e.

## 4. Discussion

Accumulating evidence underscores the critical involvement of germline mutations in the pathogenesis of CNS tumors. For instance, in Li-Fraumeni families associated with germline *TP53* mutations, it demonstrates a high predilection for CNS malignancies as one of its most frequent manifestations [23]. Similarly, Cowden syndrome, caused by germline mutations in the *PTEN* gene, predisposes individuals to CNS tumors including oligodendrogliomas, glioblastomas, and meningiomas [24]. Pathogenic germline variants in *BRCA1-Associated Protein 1* (*BAP1*) gene underlie *BAP1* tumor predisposition syndrome, characterized by a multiorgan tumorigenesis with meningeal involvement [25, 26].

The 5th edition of the WHO Classification of CNS Tumors incorporates several novel genetic tumor syndromes. Germline *elongator protein complex* (*ELP1*) mutations confer susceptibility to *SHH*-activated *TP53* wild-type medulloblastomas in pediatric populations, which are defined as “*ELP1*-medulloblastoma syndrome” [27]. *DICER1* syndrome, caused by heterozygous germline mutations in the *DICER1* gene, confers a lifetime risk of a variety of neoplastic and dysplastic lesions [28]. Melanoma–astrocytoma syndrome (MAS) is a rare autosomal dominant tumor–predisposition syndrome linked to germline mutations in the *CDKN2A* gene, characterized by an increased risk of multiple tumors, including cutaneous melanoma, astrocytoma, nerve sheath tumors, pancreatic cancer, and squamous cell carcinoma of the oropharynx [29]. Carney complex (CNC) syndrome is associated with heterogeneous pathogenic variants in *cAMP-Dependent Type I regulatory subunit alpha* (*PRKAR1A*) gene [30].

The co-occurrence of CNS and gastrointestinal pathologies is a hallmark of multiple tumor predisposition syndromes. Turcot et al. originally described the association of CNS tumors with gastrointestinal polyposis and cancers in 1959 and defined the disease as “Turcot syndrome” [31]. It has been removed from the 5th WHO edition and identified as distinct entities based on different genetic profiles, pathogenesis, and clinical features. This change highlighted the gradual shift from arbitrary and eponymic naming of the syndromes to a more gene-based system [32]. Constitutional mismatch repair deficiency (CMMRD) syndrome, caused by biallelic pathogenic variants in MMR genes *MLH1*, *MSH2*, *MSH6*, and *PMS2*, rarely *EPCAM* or *MLH3*, presents multiple CNS, gastrointestinal, and hematological malignancies [33]. Similar to CMMRD, patients with LS, who harbor a heterozygous germline mutation in MMR genes and acquire a second mutation somatically, may also develop gastrointestinal tumors and gliomas [34, 35]. CNS tumors, most commonly medulloblastoma, though relatively rare, are also reported in patients with familial adenomatous polyposis (FAP), caused by germline mutations of the tumor suppressor gene *adenomatous polyposis coli* (*APC*) [36]. The most common manifestation of FAP is hundreds of colonic polyps at a young age, and 90% of the patients developed CRC ultimately [37]. Germline *MUTYH* variants were historically identified in a fraction of *APC* mutation–negative cases with a phenotype overlapping with attenuated or classical FAP [38, 39]. Then, the function of *MUTYH* in colorectal and extracolonic malignancies as well as noncancer diseases has been reported [40].

In this study, we detected *MUTYH* germline mutations in two young adults with high-grade astrocytoma, *IDH* mutant. A novel c.467G > A (p.W156∗) *MUTYH* variant was identified in both cases. Different *MUTYH* variants have been addressed in an international database [41]. Mutations located throughout the entire *MUTYH* gene and the distribution of *MUTYH* mutations showed population differences. However, it remains controversial which variants have a critical role in tumorigenesis [42, 43]. We first reported the c.467G > A (p.W156∗) variant.

Both *MUTYH* and MMR genes play a role in DNA repair. The *MUTYH* gene encodes a DNA glycosylase involved in the BER system [2]. In humans, *MUTYH*, *OGG1*, and *MTH1* function in concert to identify and repair 8-oxoG incorporated into DNA, as well as to remove modified nucleosides [39]. In contrast, MMR proteins correct replication-associated mismatches, particularly insertion-deletion loops (indels) in microsatellite regions, which are repetitive sequences distributed throughout the human genome and consist of mononucleotide, dinucleotide, or higher-order nucleotide repeats such as (A)n or (CA)n [44]. As a result, *MUTYH* defects generate localized point mutations, with signature mutations of G:C → T:A transversions, compared to the widespread indels in MMR-deficient tumors. Mutations in MMR genes are often associated with a hypermutated phenotype, characterized by single-nucleotide variations (SNVs), microsatellite instability (MSI), and high TMB [45]. There is considerable overlap between MSI and high TMB [46]. These features confer a greater likelihood of response to immune checkpoint inhibitor therapy [47]. While *MUTYH* mutations increase oxidative damage–associated substitutions, the overall mutation count remains lower than in MMR-deficient tumors. Evidence from *Mutyh* knockout mice indicates that lack of functional *Mutyh* only modestly increases the mutation rate and less so than when there is a loss of mismatch repair [48]. Our observations in humans align with these findings from mouse models. Both cases were MSS with relatively low TMB (< 10 mut/Mb threshold currently used as a pan-cancer biomarker predicting benefit from immune checkpoint inhibition), indicating the difficulty in benefiting from immunotherapy.

*IDH* mutations were also detected in our cases. The IDH enzymes normally converted isocitrate to *α*-ketoglutarate (*α*-KG) in the tricarboxylic acid (TCA) cycle. However, the mutated enzymes converted *α*-KG to R-2-hydroxyglutarate (R-2-HG), which could be taken up by T cells and suppress T-cell activity [49]. Furthermore, *IDH* mutations induce epigenetic reprogramming of tumor cells, characterized by aberrant DNA hypermethylation [50]. In our cases, scarce immune cells in the tumor microenvironment were observed, and Case 2 showed methylation of the *MGMT* promoter, which suggested the tumor-promoting effect of *IDH* mutation, as well as potential application of IDH-targeted therapeutics and/or methylation inhibitors, rather than immunotherapy. The dual inhibitor of mutant *IDH1*/*2* enzymes, vorasidenib, received approval in the USA for the treatment of low-grade astrocytoma or oligodendroglioma with *IDH1/*2 mutations in 2024 [51].

We detected amplification of the *MET* gene in both patients. It has already been confirmed that solid tumors harboring *MET* amplification could benefit from single-agent or combination MET–directed therapy [52]. However, MET inhibitors failed to prolong the survival of patients with glioblastoma in multiple clinical trials [53, 54]. A new MET kinase inhibitor, PLB-1001, was applied in a Phase I clinical trial that enrolled *MET*-altered chemo-resistant glioma patients and achieved a partial response in at least two patients with advanced secondary glioblastoma without any significant side effects [55]. In April 2024, the National Medical Products Administration (NMPA) of China approved the use of PLB-1001 (Britinib) in patients with astrocytoma, *IDH* mutant who carried the *PTPRZ1-MET* fusion gene. MET inhibitors show promise for a wider range of applications in gliomas with *MET* alterations.

## 5. Conclusion

In conclusion, we reported a novel c.467G > A (p.W156∗) *MUTYH* variant in high-grade astrocytoma, *IDH* mutant. Integrating previous studies, we analyzed germline mutations of *MUTYH* in 11 patients with high-grade gliomas. Our findings suggested a potential association between germline *MUTYH* mutations and CNS tumor susceptibility. These observations warrant consideration of enhanced CNS surveillance in MAP patients. While our study identified germline *MUTYH* mutations in a subset of glioma patients, the small sample size precluded definitive conclusions about causality or independence from confounders. Larger cohorts with matched germline–somatic data are needed to validate this association and elucidate the mechanistic contribution of *MUTYH* in neural oncogenesis.

## Figures and Tables

**Table 1:** Summary of germline mutations in the patients.

**Case**	**Variation type**	**Gene**	**Variants**	**VAF**	**ACMG classification**
1	Germline mutation	*MUTYH*	c.467G > A (p.W156∗)	0.5075	Pathogenic
1	Germline mutation	*ERCC8*	c.197G > A (p.G66D)	0.4605	Uncertain significance
1	Germline mutation	*FANCM*	c.3308A > C (p.H1103P)	0.4942	Uncertain significance
1	Germline mutation	*KMT2A*	c.10130A > G (p.D3377G)	0.4932	Uncertain significance
2	Germline mutation	*MUTYH*	c.467G > A (p.W156∗)	0.4899	Pathogenic
2	Germline mutation	*TSC1*	c.1921C > T (p.P641S)	0.5289	Uncertain significance
2	Germline mutation	*XPA*	c.289G > A (p.V97I)	0.5464	Uncertain significance
2	Germline mutation	*ATR*	c.5899-8del (.)	0.4922	Uncertain significance
2	Germline mutation	*GLI3*	c.22 T > C (p.S8P)	0.556	Uncertain significance
2	Germline mutation	*LZTR1*	c.263+7G > A (.)	0.5469	Uncertain significance

**Table 2:** Summary of pathogenic/likely pathogenic germline *MUTYH* variants in gliomas.

**Case**	**Age**	**Gender**	**Tumor site**	**Diagnosis**	**Mutation type**	**HGVS.c**	**HGVS.p**	**VAF**	**Reference**
1	30	Female	Left frontal lobe	Astrocytoma, *IDH* mutant	Heterozygous	c.467G > A	p.W156∗	0.4899	Current study
2	38	Male	Right frontal lobe	Astrocytoma, *IDH* mutant	Heterozygous	c.467G > A	p.W156∗	0.5075	Current study
3	12	Female	Left parasagittal	Diffuse hemispheric glioma, *H3 G34V* mutant	Heterozygous	c.1178G > A	p.G393D	0.47	Bedics et al.
4	14	Male	Pons	Diffuse midline glioma, H3 K27-altered	Heterozygous	c.916C > T	p.R306C	0.42	Bedics et al.
5	6	Male	Spinal cord	Diffuse midline glioma, H3 K27 altered	Heterozygous	c.1396G > T	p.E466∗	0.49	Kline et al.
6	5	Male	Cerebellum	Medulloblastoma	Heterozygous	c.892-2A > G		0.98	Kline et al.
7	33	Male	4th ventricle	Medulloblastoma, *WNT* activated	Homozygous	c.1227_1228dup	p.Glu410Glyfs∗43	Unknown	Villy et al.
8	1.5	Female	Cerebellum	Medulloblastoma, *SHH* activated and *TP53* wild type	Heterozygous	c.1353_1355delCCT	p.Glu452del	0.54	Cipri et al.
8	1.5	Female	Cerebellum	Medulloblastoma, *SHH* activated and *TP53* wild type	Heterozygous	c.228G > T	p.Tyr76Ter	0.42	Cipri et al.
9	Unknown	Unknown	Unknown	Adult-type diffuse glioma	Unknown	c.1477G > T	p.V493F	Unknown	McDonald et al.
10	Unknown	Unknown	Unknown	Adult-type diffuse glioma	Unknown	c.1187G > A	p.G396D	Unknown	McDonald et al.
11	Unknown	Unknown	Unknown	Adult-type diffuse glioma	Unknown	c.1187G > A	p.G396D	Unknown	McDonald et al.

**Table 3:** Summary of prognostic data of patients with germline *MUTYH* mutations.

**Case**	**Age**	**Sex**	**PFS (status)**	**PFS (months)**	**OS (status)**	**OS (months)**	**Other diseases**	**Reference**
1	30	Female	1	2	0	4	None	Current study
2	38	Male	0	3	0	3	None	Current study
3	12	Female	1	6	1	6	None	Bedics et al.
4	14	Male	0	9	0	9	None	Bedics et al.
5	6	Male	1	8	1	18	None	Kline et al.
6	5	Male	1	120	0	123	None	Kline et al.
7	33	Male	1	36	0	60	Colorectal adenomatous polyposis	Villy et al.
8	1.5	Female	0	6	0	6	None	Cipri et al.

## Data Availability

All the raw data files were deposited in the Research Data Deposit (https://www.researchdata.org.cn) with the accession number RDDB2024749453.

## Nomenclature

*MUTYH*: *E. coli MutY homolog*
MAP: *MUTYH*-associated polyposis
IDH1: Isocitrate Dehydrogenase 1
CNS: central nervous system
NGS: next-generation sequencing
MMR: mismatch repair
TMB: tumor mutation burden
LS: Lynch syndrome
MSI: microsatellite instability
